# Mesenchymal Stem/Stromal Cells under Stress Increase Osteosarcoma Migration and Apoptosis Resistance via Extracellular Vesicle Mediated Communication

**DOI:** 10.1371/journal.pone.0166027

**Published:** 2016-11-03

**Authors:** Krishna C. Vallabhaneni, Meeves-Yoni Hassler, Anu Abraham, Jason Whitt, Yin-Yuan Mo, Azeddine Atfi, Radhika Pochampally

**Affiliations:** 1 Cancer Institute, University of Mississippi Medical Center, Jackson, MS, United States of America; 2 Department of Radiation Oncology, University of Mississippi Medical Center, Jackson, MS, United States of America; 3 Department of Pharmacology-Toxicology, University of Mississippi Medical Center, Jackson, MS, United States of America; 4 Department of Biochemistry, University of Mississippi Medical Center, Jackson, MS, United States of America; Universita degli Studi di Torino, ITALY

## Abstract

Studies have shown that mesenchymal stem/stromal cells (MSCs) from bone marrow are involved in the growth and metastasis of solid tumors but the mechanism remains unclear in osteosarcoma (OS). Previous studies have raised the possibility that OS cells may receive support from associated MSCs in the nutrient deprived core of the tumors through the release of supportive macromolecules and growth factors either in vesicular or non-vesicular forms. In the present study, we used stressed mesenchymal stem cells (SD-MSCs), control MSCs and OS cells to examine the hypothesis that tumor-associated MSCs in nutrient deprived core provide pro-proliferative, anti-apoptotic, and metastatic support to nearby tumor cells. Assays to study of the effects of SD-MSC conditioned media revealed that OS cells maintained proliferation when compared to OS cells grown under serum-starved conditions alone. Furthermore, OS cells in MSCs and SD-MSC conditioned media were significantly resistant to apoptosis and an increased wound healing rate was observed in cells exposed to either conditioned media or EVs from MSCs and SD-MSCs. RT-PCR assays of OS cells incubated with extracellular vesicles (EVs) from SD-MSCs revealed microRNAs that could potentially target metabolism and metastasis associated genes as predicted by *in silico* algorithms, including monocarboxylate transporters, bone morphogenic receptor type 2, fibroblast growth factor 7, matrix metalloproteinase-1, and focal adhesion kinase-1. Changes in the expression levels of focal adhesion kinase, STK11 were confirmed by quantitative PCR assays. Together, these data indicate a tumor supportive role of MSCs in osteosarcoma growth that is strongly associated with the miRNA content of the EVs released from MSCs under conditions that mimic the nutrient deprived core of solid tumors.

## Introduction

Osteosarcoma (OS) is the eighth most common type of cancer found in children and adolescents, accounting for approximately 20% of all primary bone cancers. The 5-year survival rate for osteosarcoma has increased from 20% to 70% since the 1970’s. However, for patients with metastatic disease at initial diagnosis, survival remains at 20–30% [1]. Mutations in the retinoblastoma and p53 tumor suppressor pathways and increased expression of the proto-oncogenes c-fos and c-myc are found in the majority of osteosarcomas. The insulin-like growth factor, VEGF, and transforming growth factor pathways are known to be among the key signal transduction pathways involved in OS progression. Unfortunately, despite the similar mutations and signaling pathways recognized in the disease, OS tumors have shown a large amount of heterogeneity which has made it difficult to improve the long term survival of patients with metastasis at initial diagnosis.

The tumor microenvironment has been demonstrated to play a large role in the growth and metastasis of osteosarcoma. For example, BMP-2 upregulates osteogenic markers through a Wnt-signaling dependent pathway [2] and mesenchymal stem cells (MSCs) in the tumor microenvironment produces lactate which fuels the OS cells [3, 4]. The promotion of proliferation, metastasis, and apoptosis resistance has been linked to stromal-cancer cell paracrine interactions in numerous studies. Various studies in cell culture and in xenograft models have demonstrated paracrine interactions between stromal and cancer cells that promote the proliferation and metastasis of cancer cells [5, 6]. MSCs subjected to hypoxic, nutrient poor conditions have been associated with increased secretion of tumor supportive growth factors and cytokines, including IL-6 [7] and VEGF [8], leading to decreased apoptosis and the promotion of angiogenesis. Furthermore, changes in tumor cell gene expression can be attributed to the exchange of short, non-coding 22 nucleotides RNA sequences (microRNAs) that bind to the 3’ untranslated region (UTR) of mRNAs, resulting in their silencing [9, 10]. MicroRNAs have been investigated as predictors of outcome for colorectal cancer, as promoters of breast cancer and prognostic indicators in gastric cancer [11–13]. Additionally, microRNAs have been identified as regulators of bone homeostasis and bone metastasis, making their role in osteosarcoma of considerable interest [14]. Previous publications from our laboratory showed that MSCs from bone marrow act as tumor stromal cells to support tumor cells both through paracrine and juxtacrine mechanisms [15]. Thus, the exchange of microRNAs and cytokines between cancer cells and stromal MSCs establishes a feedback loop in which MSCs in the tumor microenvironment are kept in an undifferentiated and autophagic state which feeds the cancer cells with nutrients. Extracellular vesicles (EVs) are small membrane vesicles that are released by all cell types. Specific nomenclature for EVs includes exosomes (30–100 nm diameter), microvesicles (50–1000 nm), and apoptotic bodies (50–5000 nm). Over the last several years, investigations into their therapeutic and diagnostic utility has intensified. For the purposes of these studies we used EVs that were in the size range of exosomes, which we recently characterized as containing tumor regulatory proteins, metabolites, and microRNAs [16]. In this study, we set out to characterize the role for MSCs in the growth and survival of osteosarcoma tumor growth *in vitro*. Studies using MSCs to mimic the tumor microenvironment revealed microRNAs from autophagic MSCs have the potential to alter the expression of metabolism and growth factor associated genes in surrounding tumor cells.

## Methods and Materials

### Cell culture

The human osteosarcoma cell line KHOS was obtained from ATCC. KRSOS osteosarcoma cells were from a patient derived biopsy from UMMC, Jackson, MS. The study was conducted following national and institutional ethical policies, and it was previously approved by the University of Mississippi Medical Center (UMMC) Ethical Committee and the written informed consent was obtained from all participants. The tumor was mechanically dissociated with a scalpel into 1 mm pieces, disassociated with collagenase and plated in 20 mL of DMEM (Invitrogen), 10% FBS (Atlanta Biologicals), 1% Penicillin-Streptomycin (Invitrogen). The plates were maintained at 37°C in humidified 5% CO2 and 95% air, media was changed every 2–3 days and established *in vitro*. Donor matched mesenchymal stem cells were obtained from the bone marrow at the same time as the KRSOS cells and designated as KRSBM. SD-MSCs were obtained as described previously [16]. Briefly, cells were grown to 80% confluency, washed three times with phosphate buffered saline and cultured without fetal bovine serum. The following are the different types of media used in the manuscript- Complete conditioned media (CCM): DMEM with 10% FBS and 1% Penicillin- Streptomycin; Serum deprived media (SDM): DMEM without FBS and with 1% Penicillin- Streptomycin; Conditioned media (CM): media collected from MSCs culture plates grown in SDM.

### Transwell assay

MSCs or SD-MSCs were plated in inserts of transwell permeable support 0.4 μm pore size (Corning, Lowell, MA). OS cells (10^5^ cells per well) were plated in the lower chamber of a 12-well transwell plate and incubated under standard conditions. After 24 h, osteosarcoma cells were switched to serum-free media and incubated with inserts containing MSCs or SD-MSCs for 72 h. Total OS cells DNA content was evaluated using CyQuant assay (Invitrogen, Carlsbad, CA) as described previously [17].

### Wound healing

KHOS or KRSOS cells were plated at a density at 0.5 x10^5^ cells/well in 12-well sterile, culture treated plates and incubated to near confluency at 37°C, 5% CO_2_. The cell monolayer was scraped in a straight line with a p200 pipet tip. Cells were washed once with 1 ml of the phosphate buffered saline (PBS) and then incubated with 1 ml of medium specific for the *in vitro* scratch assay. For scratch assays involving EVs treatment, EVs were isolated as previously described [16, 18], quantified by the bicinchoninic acid assay, and stored at -80°C until used. For experiments, media contained 2% serum and 50 μg EVs per 1 x 10^6^ cells. Cells were imaged every 6 hr for 24 hr and the amount of wound closure measured using ImageJ software analysis.

### Apoptosis assay

Drug sensitivity assays were done in triplicate on 24-well plates seeded at passage 3 and a density of 10^4^ cells/cm^2^. OS cells were treated with 0.1 μM doxorubicin for 24 h in the presence of SD-MSC conditioned media or alpha-Minimum Essential Medium as control. The level of apoptosis in living cells was analyzed via the red fluorescent caspase 3 and 7 fluorogenic substrate, MR-(DEVD)_2_ of the Magic Red^™^ Caspase detection kit (Immunochemistry Technologies, Bloomington, MN). Fluorescence measurements were made with a Biotek Synergy 4 microplate reader.

### Real-time quantitative PCR

Total RNA was isolated from cells using miRVana RNA isolation kit according to manufacturer’s protocol (Life Technologies, Valencia, CA). Approximately 2 μg of RNA was used in the reverse transcription reaction using the miScript SYBR Green kit (Qiagen) with random hexamers (Fermentas, Vilnus, Lithuania) according to the manufacturer's instructions. Real-time RT-PCR was performed in a Biorad myIQ thermocycler using 96-well plates. The reactions were prepared according to the standard protocol for one-step SYBR Green RT-PCR (Applied Biosystems, Cheshire, UK). The thermal cycle conditions were 95°C for 4 min followed by 40 cycles of 30 sec at 95°C, 30 sec at 55°C and 30 sec at 70°C. Averaged cycle of threshold (Ct) values of GAPDH duplicates (for genes) or RNU-6B duplicates (for microRNAs) were subtracted from Ct values of target genes to obtain ΔCt, and then relative gene expression was determined as 2^−ΔCt^.

### Statistical analysis

Statistical analysis was performed with Excel (Microsoft, Redmond, WA) and GraphPad Prism (La Jolla, CA). Statistical significance was calculated using either two-tailed Student’s *t*-test or one-way analysis of variance (ANOVA). Data is presented as the means, and error bars indicate the standard deviation. A *p*-value of <0.05 is considered to be significant.

## Results

### Serum-deprived MSCs protect OS cells from nutrient deprivation induced cell death

**Fig 1:**
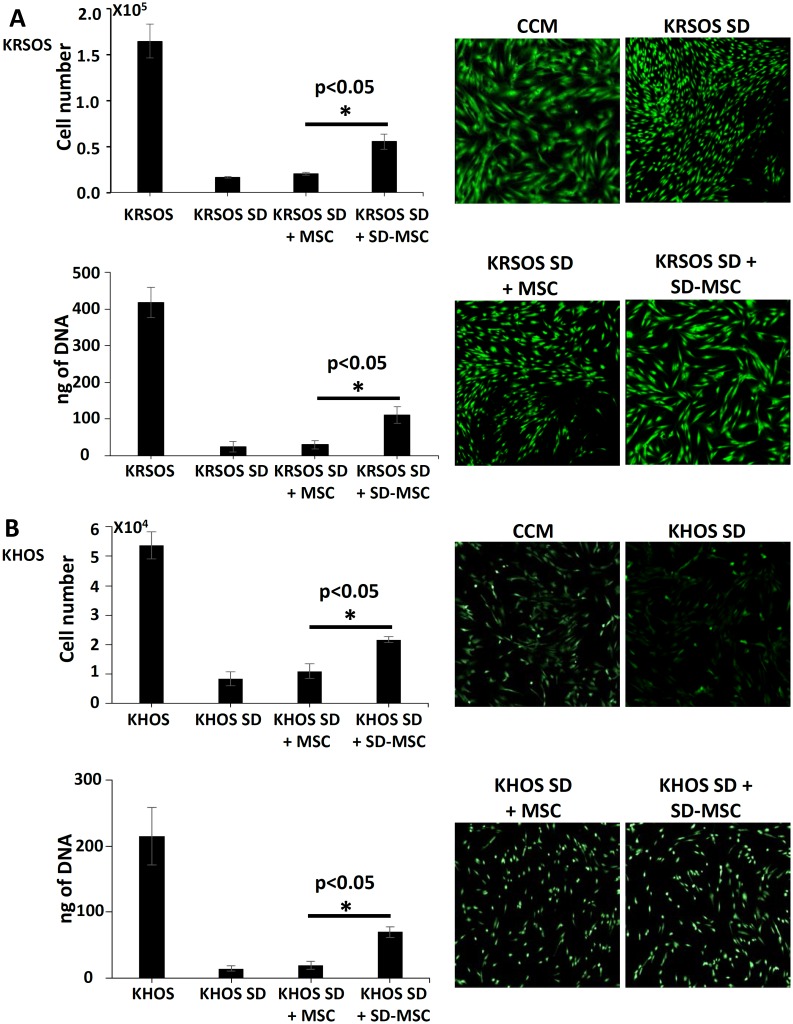
Comparative analysis of OS survival in the presence of mesenchymal stem cells (MSCs) or serum-deprived MSCs (SD-MSCs). **(A)** KRSOS cells grown with either complete culture media (CCM) or media without serum (SDM) in the presence of transwell inserts with donor-matched MSCs or SD-MSCs. **(B)** KHOS grown in either CCM or serum-free media in the presence of inserts containing either MSC or serum-deprived MSC cells. Data presented as means ± SD, Columns, mean of three independent experiments; bars, standard deviation (SD).

Osteosarcoma cells placed in serum-deprived conditions showed a significant decrease in both cell number and DNA content after being cultured in serum-free conditions for 72 h (Fig 1A and 1B). The cell number of KRSOS cells grown in the presence of SD-MSC inserts was more than 3-fold higher than OS cells grown without serum, but there was only a 20% increase in the cell number of serum-deprived OS cells grown in the presence of inserts containing MSCs (Fig 1A). DNA content measurement followed the same pattern as that observed with cell number. Similarly, the presence of SD-MSC inserts restored KHOS cell viability by more than 2-fold as measured by direct cell counting and more than 3-fold by DNA quantification (Fig 1B). As observed with KRSOS cells, there was no significant increase in KHOS survival when grown in the presence of MSCs, supporting the hypothesis that SD-MSCs release the growth factors and microRNAs that support OS growth.

### SD-MSC conditioned media protects OS cells against drug induced apoptosis

**Fig 2:**
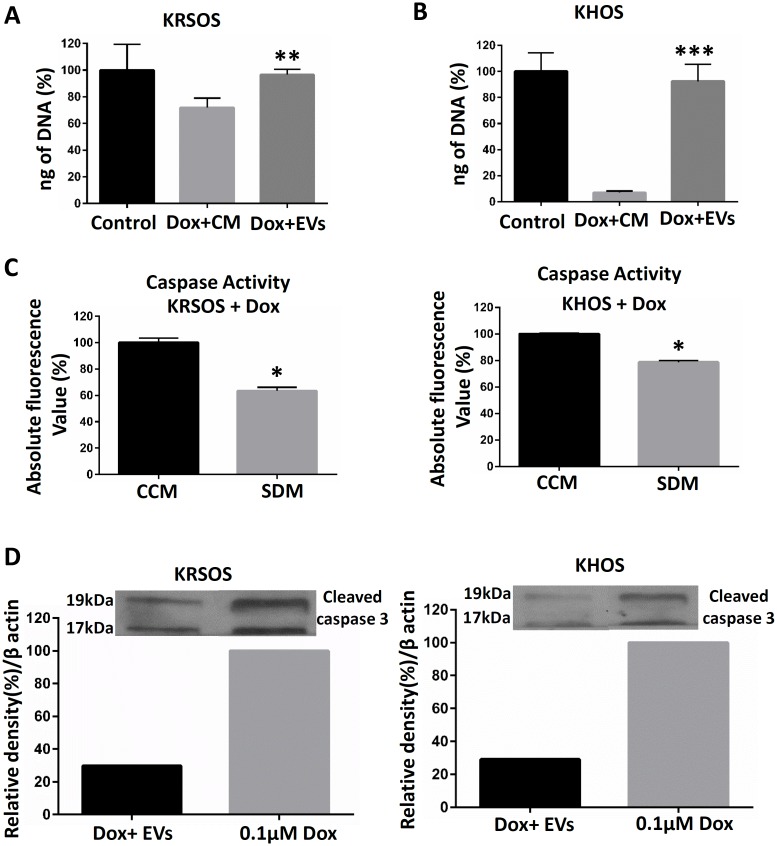
Apoptosis analysis of OS cells incubated with conditioned media from serum-deprived MSCs. **(A and B)** DNA quantification of KRSOS and KHOS osteosarcoma cells treated with 0.1 μM doxorubicin for 24 h in the presence of complete culture media (control), SD-MSC media (CM) or EVs from SD-MSCs (EVs). **(C)** OS cells treated with doxorubicin in the presence of either SDM, CCM. Caspase activity measured as an increase in the amount of fluorogenic DEVD_2_, a caspase 3 substrate. The data presented as the means ± SD of 3 independent experiments, *P < 0.05. **(D)** Western blot of cleaved caspase-3 in KRSOS and KHOS treatment with doxorubicin in the presence of SD-MSC EVs.

As a second approach to test for the role of pro-survival factors present in the serum-deprived supernatant of MSCs, osteosarcoma cells were incubated in the presence of 0.1 μM doxorubicin and grown in the presence of conditioned media from SD-MSCs (CM). In the presence of doxorubicin plus conditioned media and doxorubicin plus EVs, KRSOS exhibited a close to 100% and KHOS exhibited a 80% increase in cell survival compared to KRSOS and KHOS cells in the presence of doxorubicin in complete culture media (CCM) respectively (Fig 2A and 2B). Furthermore, caspase activation was significantly decreased in both KRSOS and KHOS cells treated with doxorubicin (Fig 2C and 2D) in the presence of SDM. These data indicate media from SD-MSCs have anti-apoptotic properties.

### SD-MSC conditioned media and SD-MSC derived EVs increase wound healing in OS cells

**Fig 3:**
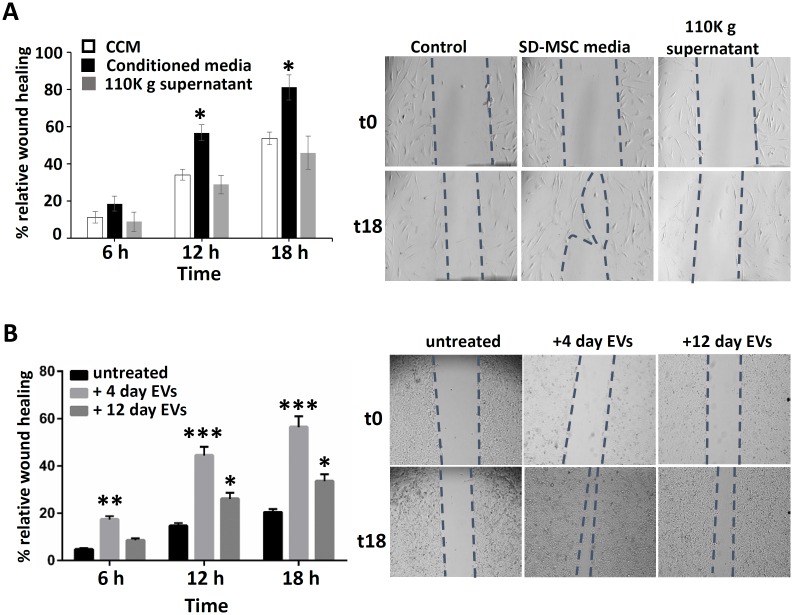
Effects of EVs and conditioned media from SD-MSCs on OS wound healing. **(A)** KHOS cell monolayer was scratched with a p200 micropipette tip, incubated with CCM or SD-MSC conditioned media, and imaged every 6 hrs, after replacing the media with serum free media, SD-MSC conditioned media or EV free media. **(B)** Scratched KHOS cell monolayer treated with EVs from SD-MSCs for either 4 days or 12 days and imaged every 6 hrs after replacing the media with SDM + EVs. Wound closure area was determined by ImageJ software analysis. Data presented as the means of three independent measurements. * P< 0.05, ** P< 0.01, *** P< 0.005 compared to untreated controls.

The increase in the metastatic potential of osteosarcoma and other cancers had been previously attributed to soluble factors secreted by MSCs [19–21], yet few studies had examined them. We determine the wound healing rate of OS cells incubated either with SD-MSC media or EVs isolated from SD-MSC media. Compared to control, the rate of wound closure in KHOS cells exposed to SD-MSC conditioned media or 110k supernatant (that is EV free) was greater at the 6, 12, and 18 h time points, respectively, congruous with previous reports (Fig 3A). Correspondingly, EVs from MSCs (4days) and SD-MSCs (12days) increased the wound closure rate of KHOS cells by more than 50% of the relative amount at those time points (Fig 3B). This data supports previous studies backing the hypothesis that EVs transport tumor regulatory microRNAs and metabolites [16, 22].

### Would healing property of EVs is associated with altered expression of microRNAs and gene targets

**Fig 4:**
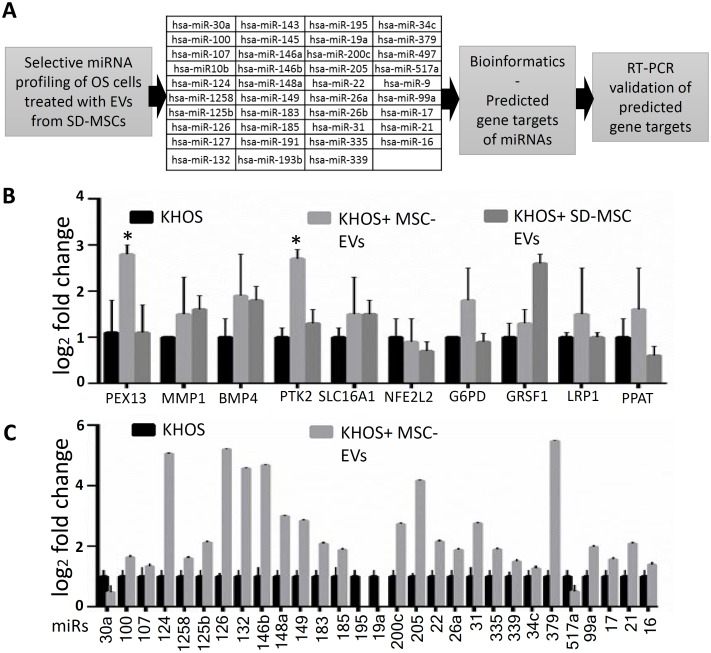
**(A)** Schematic procedure for determining microRNAs and genes associated with changes in OS cells after treating with to EVs from MSCs (4day EVs) and SD-MSCs (12day EVs). **(B)** Quantitative RT-PCR validation of changes in metastasis and metabolism associated genes after OS incubation with MSCs and SD-MSC-EVs. **(C)** Quantitative RT-PCR showing changes in expression of shortlisted miRNA after MSC-EVs treatment.

Some studies have shown that microRNAs play an important role in osteoblast differentiation and bone formation [23–25]. Furthermore, microRNAs can be transferred between cells via EVs and several microRNAs have been implicated in osteosarcoma growth [26–28]. In consideration of the potential contribution of microRNAs to OS growth, we investigated the common downstream targets and the underlying molecular mechanisms of tumor growth supportive miRNAs. A schematic for data analysis is shown in Fig 4A and 39 miRNA with potential role in tumor growth were short-listed for further analysis. To further narrow down the target genes we used in silico algorithms (TargetScan, PicTar, miRanda) to predict common target genes of the short listed 39 miRNA. A number of genes were targeted by these microRNAs from which 21 genes were selected for further study. We then used quantitative RT-PCR analysis to validate genes identified as possible targets of the microRNAs (Fig 4B), focusing on genes associated with metastasis and cellular response to nutrients. Quantitative RT-PCR assay to confirm the expression of 29 of the 39 short listed miRNA following bioinformatics assays is shown in Fig 4C. The microRNA with one of the higher differential expression, *hsa-miR-148a*, has been identified as a prognostic biomarker in osteosarcoma patients, with high expression levels correlating with decreased overall survival [29].

**Fig 5:**
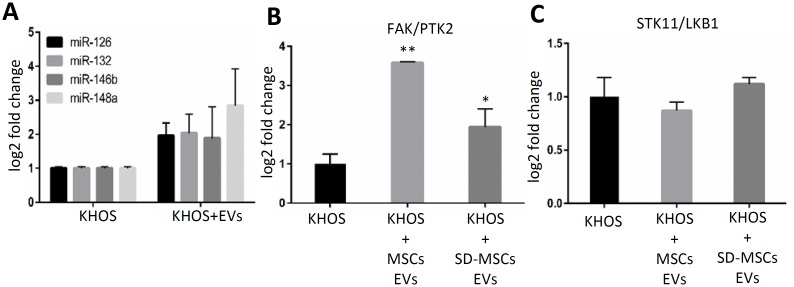
miRNA transferred by EVs regulate PTK2 and expression. **(A)** Quantitative RT-PCR showing changes in expression of four miRNA after MSC-EVs treatment. **(B)** Quantitative RT-PCR expression of downstream target PTK2/FAK. **(C)** Quantitative RT-PCR showing downregulation of FAK1repressor LKB1.

Next as a proof of concept we have tested expression of a cluster of four miRNAs with potential binding sites that regulate the pathway of focal adhesion point kinase1 or PTK2 (Fig 5A). One gene that is ontologically connected to metabolism (PTK2) and 4 microRNAs that have putative targets in LKB1 /PTK1 and STK11/FAK1 pathway. Liver kinase β1 (LKB1, also known as STK11) is a serine/threonine kinase that has multiple cellular functions including the regulation of cell polarity and motility. It is shown that LKB1 represses FAK signaling via a FAK-LKB1 complex to regulate FAK site maturation. Experimental validation of predicted miRNA targets revealed an increase in the expression of focal adhesion kinase (PTK2) genes (Fig 5B), implicating EV-mediated miRNA transfer in high grade, aggressive osteosarcomas, in agreement with previous observations [30, 31].

**Fig 6:**
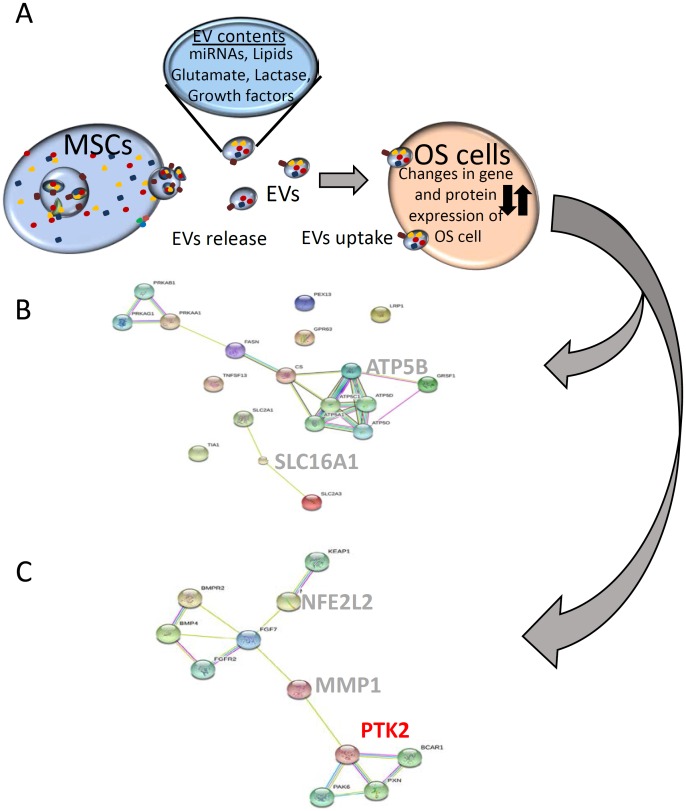
**(A)** A simplified interaction schema between EV-derived miRNAs and changes in gene expression in osteosarcoma cells **(B)** Metabolic pathways involved in osteosarcoma metastasis highlighting the lactate importer MCT1 (SLC16A1) and oxidative phosphorylation pathways (ATP5B). **(C)** Cell migration pathway in osteosarcoma associated with exposure to EV miRNAs emphasizing the interaction between the focal adhesion (PTK2) and oxidative stress pathways (NFE2L2).

Another significant observation was the slight decrease in the gene expressions of NFE2L2 (Nrf2, an important mediator in the ability of cells to adapt to oxidative stress. Additionally, increased SLC16A1 (MCT1) expression and increased *GRSF1* expression have been previously shown to decrease doxorubicin sensitivity and attenuate oxidative phosphorylation, respectively [32] [33] (Fig 6B). The decreased expression of NFE2L2 is characteristic of the paradoxical role of NFE2L2 in cancer progression. Decreased levels would be expected to increase tumor cell susceptibility to apoptosis, yet NFE2L2 deficiency may increase the risk of pulmonary metastasis [34] although the significance of decreased expression of the gene in osteosarcoma is not well understood. However, reactive oxygen species are known mediators of PTK2 activation. In fact, the regulation of NFE2L2, and PTK2 expression are linked indirectly through FGF7, a member of the fibroblast growth factor family involved in wound repair and tumor development [35] [36] (Fig 5C).

## Discussion

The ability of SD-MSC conditioned media and EVs to increase survival and reduce apoptosis correlates with previously published results demonstrating that EVs from MSCs can supply proteins, metabolites and microRNAs to support tumor growth [16]. Several studies have shown that tumors have the ability to manipulate their stromal environment via the transport of microvesicular contents, including interleukins, monocyte chemotactic protein-1 (MCP-1), macrophage inflammatory protein- 1 beta (MIP-1β), and chemokines [37]. These microenvironmental changes can influence tumor progression as well as the effectiveness of chemotherapy. Such changes may even inhibit the growth of normal cells, promote the growth, and clonal expansion of neoplastic progenitor cells. Once established, tumor cells can inhibit the differentiation of mesenchymal stem cells in the surrounding stroma and induce them to produce cytokines and other factors that support tumor growth [15]. While the release of metabolites and cytokines all undoubtedly play a role in tumor stroma interaction, our current understanding of tumor-stroma co-evolution is far from complete due to the number of different metabolites, cytokines and microRNAs in the tumor microenvironment.

In the present study, comparison of the effects of MSC media and derived EVs revealed similar effects of the two treatments on OS survival. Our results revealed significant downregulation of *hsa-miR-195* and *hsa-miR-124* in parallel with an upregulation of *hsa-miR-148a*. Consistent with these results, Cai *et al*. have previously reported *hsa-miR-195* levels in sera from osteosarcoma patients were significantly lower than those in healthy controls [38], and *hsa-miR-124* overexpression has been shown to inhibit migration in MG-63 and U2OS cells [39]. Additionally, the increased expression of *hsa-miR-148a* is significantly associated with tumor size and distant metastases [29]. RT-PCR validation of predicted miRNA targets revealed an increase in the expression of matrix metalloproteinase (MMP1) and focal adhesion kinase (PTK2), implicating EV-mediated miRNA transfer in high grade, aggressive osteosarcomas, in agreement with previous observations [30, 31]. The association between PTK2 gene expression and EV treatment in these experiments correlates with the known role of PTK2 in cell migration and supports previous studies pinpointing the importance of PTK2 expression on patient survival [40]. The increase in cyclin D1 expression in parallel with PTK2 and MMP1 expression strongly suggests a specific connection between decreased *hsa-miR-195* levels and OS aggressiveness. More generally, comparison of the differentially regulated genes with a database of known and predicted protein interactions confirmed metastasis, oxidative stress, and metabolic signaling pathways were involved (Fig 6B and 6C). Increase in the expression of the monocarboxylate transporter, SLC16A1 adds to the hypothesis. These data reveal OS cells exposed to SD-MSCs increase their ability to uptake lactate, which has been associated with poorer clinical outcomes [41]. The migratory response of cancer cells has also been correlated with increased SLC16A1 (MCT-1) expression [42]. The convergence of the oxidative stress and focal adhesion pathways as shown in Fig 6 are consistent with previous observations associating cell motility and tumor aggressiveness with reactive oxygen species intermediates [43] and are emblematic of the delicate balance between redox signaling, healthy cells, and pathology.

Our results have shown that cell-to-cell communication via EVs from SD-MSCs can significantly affect the metastatic potential of OS cells. Although several studies have identified microRNAs and target genes that can be associated with osteosarcoma metastasis, few have studied the implications of tumor-associated mesenchymal stem cells on the metastasis and prognosis of OS. Our data has correlated specific microRNA with downregulation of predicted gene targets and is consistent with previous studies which have shown that EVs, <100nm vesicles secreted by cells, can significantly affect the growth characteristics of cells in close proximity to the secreting cell. However, there are numerous examples of miRNAs repressing translation of endogenous mRNAs without appreciably affecting the mRNA level [44, 45], which may confound RT-PCR analysis in which miRNA expression does not correlate with gene expression. Additionally, comparison of only two cell lines that do not allow us to generalize the contribution of the tumor stroma to the progression of OS as there are examples of MSCs inhibiting growth in other tumor types [46–48]. Further studies will be required to determine if miRNAs can be transferred in physiologically relevant amounts to account for these results or if they can be attributed to other components secreted within EVs.
